# A mixed-methods study of women’s sanitation utilization in informal settlements in Kenya

**DOI:** 10.1371/journal.pone.0214114

**Published:** 2019-03-21

**Authors:** Samantha Cristine Winter, Robert Dreibelbis, Millicent Ningoma Dzombo, Francis Barchi

**Affiliations:** 1 Edward J. Bloustein School of Planning & Public Policy, Rutgers, The State University of New Jersey, New Brunswick, New Jersey, United States of America; 2 Department of Disease Control, London School of Hygiene and Tropical Medicine, London, United Kingdom; 3 Department of Sociology and Social Work, University of Nairobi, Nairobi, Kenya; Tulane University School of Public Health and Tropical Medicine, UNITED STATES

## Abstract

While access to safe sanitation is a global issue, there are large disparities in access. Women living in informal settlements, in particular, are disproportionately affected by lack of access to sanitation. Without adequate sanitation, these women may resort to unsafe strategies to manage their sanitation needs, but limited research has focused specifically on this issue. Qualitative and quantitative data were collected from women in the Mathare informal settlement in Nairobi, Kenya in 2016. A latent class analysis (LCA) using the quantitative data yielded five distinct sanitation profiles (SP) among women in Mathare. In-depth interviews and sanitation walks with women added further detail about the characteristics of and motivations underlying each profile. Women’s sanitation profiles in these settlements are complex. A majority of women in this study utilized an unsafe method of disposal at least once in a 24-hour period that increased their risk of direct exposure to waste and harmful pathogens.

## Introduction

Despite increasing global efforts to expand sanitation coverage, 2.4 billion people lack access to safe sanitation facilities around the world [1]. The health consequences of lacking access to safe sanitation are many [2–4], including diarrheal disease—one of the leading causes of death in children under five—as well as other tropical diseases such as cholera and typhoid and parasitic infections including Guinea Worm Disease, Buruli Ulcer, Trachoma, and Schistosomiasis [5–7]. While access to sanitation is a global issue, there are large disparities in access across different regions, countries, and social and geographical contexts [8]. Nearly half of the population in developing countries, for example, has suffered from diseases associated with poor sanitation [7, 9, 10]. Lack of access to sanitation is a persistent problem in sub-Saharan Africa, where less than 20% of the current population have access to safe sanitation [1].

The problem of lack of adequate sanitation is even more critical for people living in informal settlements in sub-Saharan Africa where sanitation coverage rates are not only lower for these poor, urban settlers (e.g. 42% of the urban poor in SSA use improved or shared sanitation compared to 91% of wealthy urban dwellers), but the risk of spreading communicable diseases such as cholera and dysentery is higher. For example, prevalence of diarrhea among the children under 3 in informal settlements in Nairobi is around 32% compared to 13% in the rest of Nairobi) [2, 11–13]. Lack of adequate sanitation has been cited as one of the top concerns in Kenya, particularly in informal settlements. where, according to the World Health Organization, cholera outbreaks occur every year with most cases originating in Nairobi, and cyclical epidemics of cholera lasting 2–3 years occur every 5–7 years [14]. In addition, recent estimates suggest poor sanitation results in serious economic losses in these settings. Nairobi County, for example, loses approximately 1.7 billion Kenyan shillings (about US $17 million) each year due to poor sanitation [15].

The burden of lack of access to sanitation in informal settlements falls disproportionately on women [16]. Women are often the most vulnerable to the effects of poor sanitation partly because of their biology (e.g. menstruation and pregnancy) and partly because they are, due to their lower social and economic status, less likely to have access to good sanitation and hygiene [17, 18]. Findings from one study carried out in Mathare Valley Informal Settlement (Mathare) in Nairobi, Kenya revealed that almost 30% of female respondents reported at least one case of diarrhea in the month leading up to the study [16]. Absent private and hygienic toilets, women also suffer from urinary tract infections and hemorrhoids due to urine and feces retention and, during menstruation, increased risk of infection and toxic shock syndrome [19–22]. Lack of access to sanitation may also be associated with other less studied health consequences for women. For example, women without access to adequate sanitation are forced to use unsafe facilities or walk long distances, often at night, to find a place to relieve themselves, thereby increasing their vulnerability to physical and sexual assault [22, 23].

Access to safe sanitation can improve public health and save lives in the least expensive and most efficient way [24]. Water, sanitation and hygiene interventions reduce the incidence of water-borne and communicable diseases—often yielding widespread health improvements for the whole community [16]. However, improvements to sanitation require knowledge about the state of existing access to sanitation [25]. According to the literature, many of the recent solutions and interventions to the persistent lack of access to sanitation have not been as effective as intended largely because they fail to consider the specific sanitation needs and utilization practices of women and girls [26, 27].

Despite recent efforts to explore women’s unique sanitation behaviors in informal settlements [16, 18, 19, 28, 29], much of this evidence is anecdotal or exclusively qualitative. In addition, while recent literature has started to suggest that women's sanitation utilization differ between night and day, particularly in informal settlements, [26, 30], most studies continue to ask questions only about people’s primary sanitation facility. The aim of this research, therefore, was to develop a more nuanced understanding of women’s daily and nightly sanitation practices in informal settlements in Nairobi, Kenya. In particular, this study sought answers to the following research questions: 1) how do women manage their sanitation needs differently during the daytime and the nighttime? 2) is there a general pattern to women’s sanitation utilization, and 3) what might be the underlying reasons for this pattern? Since women in the Mathare Valley Informal Settlement, the site for this study, use the term “short call” to refer to urination/urine disposal and “long call” for defecation/disposal of feces, we, too, will use these colloquial terms for the remainder of the paper.

## Methods

**Fig 1:**
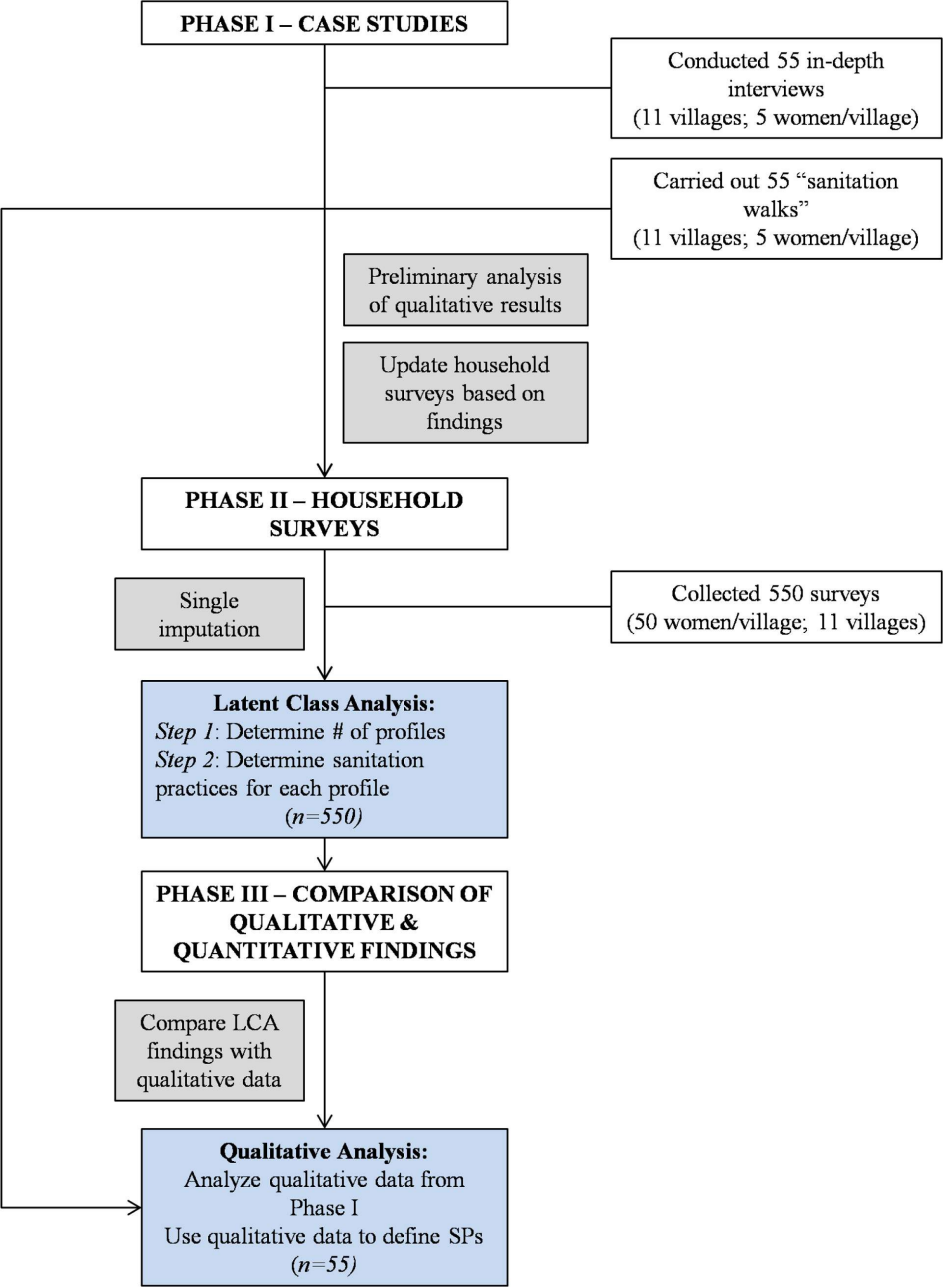
Flow diagram of data collection and analysis.

Data for this study were collected in 11 villages that make up the Mathare Valley Informal Settlement in Nairobi, Kenya. Mathare is one of the oldest informal settlements in Kenya and one of the largest informal settlements in East Africa. In informal settlements in Nairobi, Kenya, where more than half of the city’s population lives, access to improved sanitation is severely lacking [16, 26, 28, 31]. Estimates suggest that over 68% of the residents in Nairobi’s informal settlements use shared toilet facilities and an additional six percent have no access to toilets at all—relying on open spaces, “flying toilets” (plastic bags), or buckets as their primary means of meeting daily sanitation needs [31, 32]. Sampling for this study was stratified across villages. Also, because of the exploratory nature of this study, a three-phase, mixed methods approach was used to guide data collection and analysis. See Fig 1 for a summary of the phases of data collection and analysis.

*Phase I* involved: 1) in-depth interviews with 55 women (5 from each village), aged 18 years and older, purposefully sampled from each of the 11 villages, and 2) “sanitation walks” with women in their communities (data collected included photographs, field notes, observation checklists, and geospatial information). Since this study focused primarily on sanitation, participants were iteratively selected to represent a broad range of sanitation practices (e.g. open defecation; flying or bucket toilets; public facilities; shared, private facilities; and private sanitation facilities). We discovered early on in the research process that available sanitation alternatives vary across villages in Mathare due to differences in geography; accessibility; governmental, non-governmental, and private infrastructure investments; population densities; and land ownership. Thus, we also stratified the sample—making sure to include 5 women from each village. Participants were asked to give written consent for each component of the study separately (interviews, sanitation walks, GPS data, audio recordings, and photographs). Interviews and sanitation walks were conducted in English or Swahili. Interviews took place in women’s homes and lasted 40–90 minutes and walks lasted 30–60 minutes. Preliminary analysis of data from this phase helped improve data collection in Phase II and was used in Phase III to further define sanitation profiles.

Phase II involved collection of 550 household surveys. The sample was stratified across Mathare’s 11 villages because of the variability of access to sanitation between villages. Households were randomly selected within each village using the fishnet and point sampling tools in ArcMAP [33]—a technique that has been employed to assist in random, household selection in other studies in informal settlements [34]. Surveys were administered to one woman in each household who was selected using Kish methodology [35]. A field staff of 11 women (one from each of Mathare’s villages) was drawn from among the sample of women interviewed in *Phase I* to work with the research team to administer the surveys. All participants were asked to provide oral consent. Oral consent was deemed appropriate by the Internal Review Board at Rutgers because a written consent document would have been the only identifying piece of information linking otherwise anonymous surveys to participants (US regulation 45 CFR 46.117). If participants consented, the interviewer signed a document affirming consent was given. The questionnaires were read out loud to participants in either English or Swahili and filled in using paper surveys. Surveys lasted approximately 40–60 minutes. Data from this phase were analyzed prior to Phase III in order to develop general sanitation profiles.

*Phase III* was the analysis phase; descriptions and explanations relating to the sanitation utilization practices reported by women in the qualitative sample were used to verify and provide a richer context in which to understand utilization profiles that were identified in the analysis of the quantitative data. The combination of these data enabled the study team to create profiles representing common daily strategies for sanitation used by women in Mathare.

### Measures

Questions for the qualitative portion of the study were broad—allowing women to speak openly about their sanitation utilization choices. Women were asked, for example, to describe their daily and nightly sanitation routines for short and long calls, separately. Follow-up questions and probes were pursued if the women were confused or did not provide enough detail about the type and nature of their toilet/method of disposal. Questions on the survey were modified to account for these potential differences in sanitation practices during the day and at night and for short and long calls, separately. Four nominal variables (i.e. 1. long calls, day; 2. short calls, day, 3. long calls, night; 4. short calls, night) with six categories each (1. private toilet; 2. private toilet-shared between more than one household; 3. plot toilet; 4. bags/buckets in the home; 5. public toilet and 6. no facility/open defecation outside the home) were created from responses to the following survey questions: “what kind of toilet or method of disposal do you usually use for short call/long call during the day/night?” Responses included, flush, pour flush; ventilated improved pit latrine (VIP); pit latrine with slab; pit latrine without slab/open pit; composting toilet; bucket toilet; hanging toilet/hanging latrine; plastic bag; no facility, bush or field; other (specify). Responses from follow-up questions were then used to identify the location of the facilities (e.g., in a house, plot, or building) and whether or not these were private, shared with other households, or public.

### Analysis strategy

This analysis utilized responses from both the quantitative (550 surveys) and qualitative (55 case studies) data. There was minimal missing data on the responses to the questions pertaining to this analysis (less than one percent). Random, single-response imputation was used to fill in the values for the quantitative data using the user-written program hotdeckvar in Stata v.14 [36].

The first stage in the analysis was to run a two-step latent class analysis (LCA) with data from all 550 quantitative surveys. The purpose of the LCA was to develop a set of common sanitation utilization profiles for women in informal settlements in Kenya. Analysis was carried out using the University of Pennsylvania’s doLCA plugin in Stata. The four nominal variables created to represent women’s sanitation facility or short call/long call methods during the day and night were used as the primary indicator variables for the LCA. The first step of the two-step LCA was to determine the appropriate number of factors in the LCA and the second step was to determine the common sanitation profiles (SPs) for women in Mathare. A covariate for village (a nominal variable with 11 categories for each village in Mathare) was used in the model to control for the stratified nature of the sampling method.

Atlas.ti software was used to carry out cross-case analysis of responses from the full transcriptions of the 55 qualitative interviews. A modified grounded theory approach was used to guide the collection and analysis of qualitative data in this study. We used an iterative process—allowing women's day-to-day experiences with sanitation to guide the multiple stages of data collection, coding, and analysis. Transcripts from the 55 in-depth interviews were coded, independently, by three researchers. All women in the study were asked to describe their primary and alternative sanitation management strategies for urination and defecation during the day and during the night, separately, and their reasons for adopting and using those strategies. A list of pre-defined codes and sensitizing concepts, based on the sanitation-related questions and response categories in the quantitative survey (see measures section) and the researchers’ experiences conducting interviews and sanitation walks in the field was developed to help guide the analyses of the qualitative responses; however, each coder added his/her own individual concepts and codes as they iteratively reviewed and compared the transcripts to help provide a deeper description of each participant's primary sanitation management strategies throughout a 24-hour period and their reasons behind their use of those strategies. Codes, concepts, and themes were then compared among the three researchers. The comparison of the codes, concepts and themes related to women’s current sanitation practices and their reasons for using those practices yielded 97% agreement between the three researchers. In instances where the codes did not agree, the full research team discussed discrepancies and agreed upon final codes and resulting findings. Finally, a group of 11 women (1 from each village) reviewed the findings and results from the study and confirmed that they aligned with their own perceptions of women's sanitation management strategies in Mathare.

### Ethics statement

The study protocol underwent ethics review and received research permits from the Kenya’s National Commission for Science, Technology, and Innovation and the Institutional Review Boards at Rutgers University, New Brunswick.

## Results

### Descriptive statistics

Descriptive statistics of the full quantitative sample (n = 550) are summarized in Table 1. The average age of the respondents in this study was 32 years (SD = 8.4), with a range of 18–70 years. Approximately 45% of the respondents had completed primary school with no or some secondary education, and approximately 31% of the women in the study completed secondary education. About 37% of the women were employed at the time of the survey, and about 23% reported owning a business. Although 54% of the women were legally married at the time of the survey, over 57% of the women in the sample reported that they were living in households headed by a female.

**Table 1:** Descriptive statistics of quantitative and qualitative samples.

	Quantitative (n = 550)		Qualitative (n = 55)	
	Frequency	Percent	Frequency	Percent
Number of children				
None	101	18.4	1	1.8
1–2 children	247	44.9	29	52.7
3–4 children	155	28.2	14	25.5
5–6 children	41	7.5	4	7.3
7+ children	6	1.1	4	7.3
Age				
18–24	85	15.5	3	5.5
25–34	300	54.5	25	45.5
35–44	116	21.1	17	30.9
45–55	36	6.5	4	7.3
55+	13	2.4	4	7.3
Monthly income				
Less than 5,000 Ksh/month	136	24.7	14	25.5
5000–10,000 Ksh/month	275	50	17	30.9
10,000–15,000 Ksh/month	103	18.7	3	5.5
Over 15,000 Ksh/month	31	5.6	6	10.9
Does not know	5	0.9	13	23.6
Education				
None	11	2	1	1.8
Some primary, not complete	91	16.5	12	21.8
Completed primary	136	24.7	18	32.7
Come secondary, not complete	121	22	12	21.8
Completed secondary	172	31.3	9	16.4
Higher education	17	3.1	1	1.8
Marital status				
Married	297	54	26	47.3
Living with a man, not married	11	2		
Regular partner, live apart	46	8.4		
Not involved in a relationship	191	34.7	15	27.3
Separated or divorced			11	20
Respondent has a business	126	22.9	31	56.4
Respondent is employed	205	37.3	19	36.4
Lives in female-headed household	315	57.3	29	52.7

Descriptive statistics for the qualitative sample (n = 55) are also summarized in Table 1. About 50% of the respondents in the sample were between the ages of 25 and 34 years—ranging from 18–72 years. About 17% of the sample reported having completed secondary school, while about 57% reported having completed primary school or primary and some secondary school. Over 60% of the women in the sample reported that they were not formally employed; however, about 17% reported having odd jobs (e.g. contract work or housework for a wealthier family) and almost 59% reported having some form of informal business (e.g. selling vegetables, fries, phone credit, or second-hand clothes). About 50% of the women who were interviewed were married.

#### Sanitation utilization descriptive statistics

**Fig 2:**
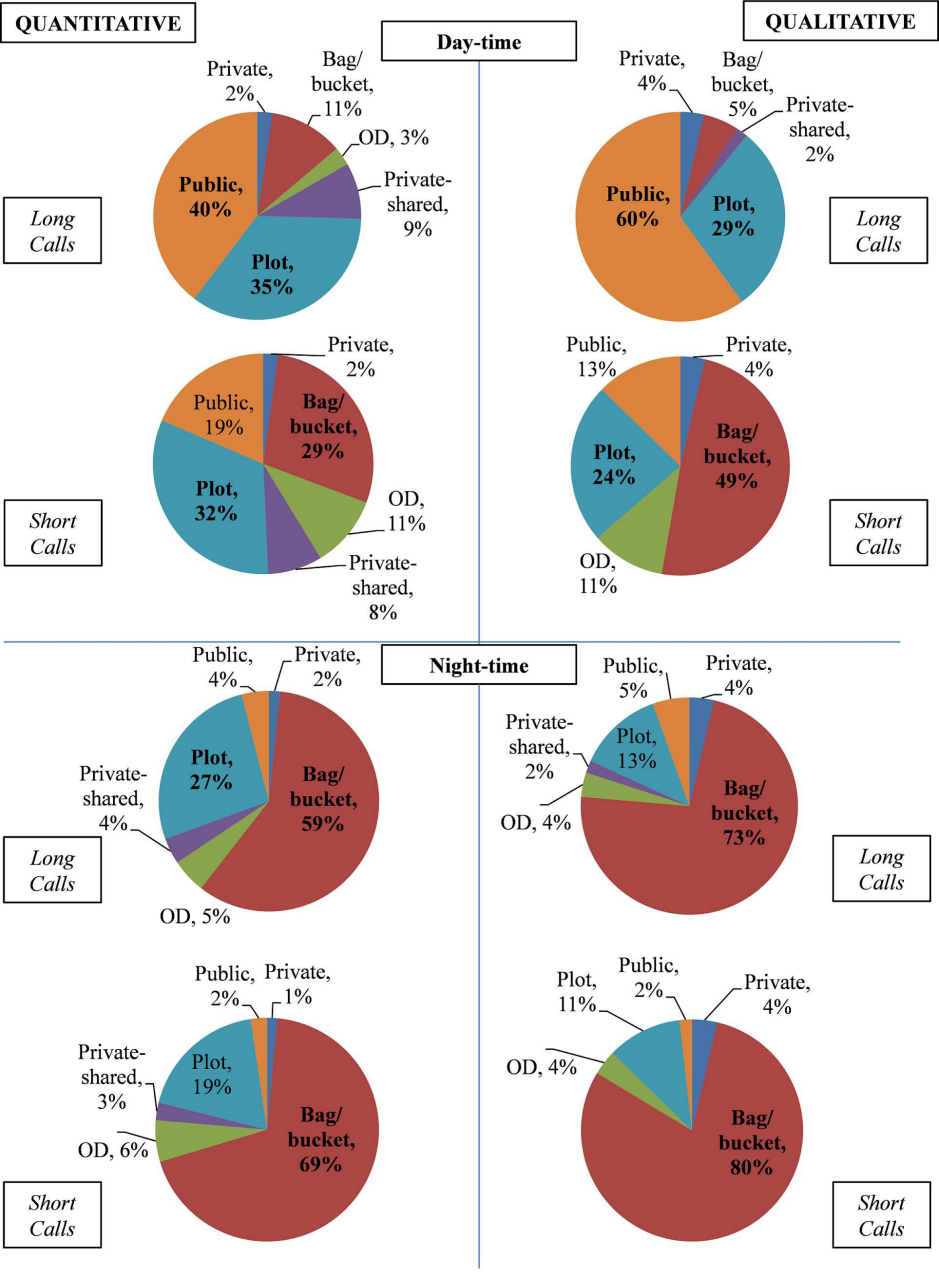
Sanitation utilization for quantitative and qualitative samples.

Frequencies for women’s reported sanitation utilization patterns in both samples are summarized in Fig 2. About 40% of women in the survey sample and about 60% of the women in the qualitative sample reported using a public toilet facility as their primary long call method during the day. An additional 35% of women in the survey sample and 39% of the qualitative sample reported using plot toilets for long calls during the day.

Very few women, only 14% of the survey sample and only 5% of the qualitative sample reported using bags, buckets, or open defecation for long calls during the day. However, the proportion of women using bags, buckets, or open defecation for short calls during the day was much higher in both the quantitative (39%) and qualitative samples (59%). At night the majority of women switched to using bags, buckets, or open defecation for short and long calls in the quantitative (64% for long calls; 75% for short calls) and qualitative (77% for long calls; 84% for short calls) samples.

### Quantitative results

#### Sanitation utilization profiles

The first step in the LCA was to determine the number of profiles based on model fit statistics. Model fit statistics are summarized in Table 2. The CAIC and BIC suggest that the 5-profile model was the best fit for the data—with values for the CAIC and BIC decreasing for the 2–5-profile models and increasing for the 6-profile model. The entropy-squared values also suggest the 5-profile model was the best fit for the data.

**Table 2:** Comparison of 1–6 profile LCA models.

	1-profile	2-profiles	3-profiles	4-profiles	5-profiles	6-profiles
df	1275	1254	1233	1212	1191	1170
Entropy-squared	NA	0.975	0.967	0.984	0.989	0.977
Adjusted BIC	2486	1498	1306	1038	886	837
CAIC	2570	1670	1565	1385	1320	1359
BIC	2550	1629	1503	1302	1216	1234
AIC	2463	1452	1236	944	768	696
G-squared	2423	1370	1112	778	560	446
Log-likelihood	-2785	-2258	-2129	-1962	-1853	-1796

Sanitation utilization profiles based on the 5-profile LCA are summarized in Table 3. Findings suggest that there are five common sanitation utilization profiles for women living in Mathare. A majority of the women who have a high probability of being in the first profile use public or plot toilets for long calls and short calls during the day and use bags/buckets in or OD near the home for both long and short calls during the night. A majority of the women who have a high probability of being in the second profile use a public toilet during the day for long calls, and use bags/buckets or OD for short calls during the day and for both long and short calls during the night. The majority of women who have a high probability of being in the third profile use a plot toilet for all long and short calls during the day and night. A large proportion of the women who have a high probability of being in the fourth profile use a private-shared, private or plot facility for long and short calls during the day and for long calls during the night; however, the majority of these women use bags/buckets for short calls during the night. Finally, women who have a high probability of being in the fifth profile mostly use bags/buckets in or OD near the home for all long and short calls during the day and for long and short calls during the night.

**Table 3:** Sanitation utilization profiles based on 5-profile LCA using quantitative data.

		Long call	Short call
Profile 1 –Lack of security at night (6%)	Day	Public (48.5%); OD (27.2%); Plot (15.3%); Bags/bucket (6.0%); Private-shared (3.1%)	OD (45.1%); Public (42.3%); Plot (9.2%); Private-shared (3.1%); Bags/buckets (0.2%)
	Night	OD (75.3%); Bags/bucket (18.3%); Plot (3.3%); Public (3%)	OD (99.2%); Bags/buckets (0.6%); Plot (0.2%)
Profile 2 **–**Lack of funds and accessibility (32.8%)	Day	Public (97.5%); Plot (2.4%); Private-shared (0.1%)	Public (43.0%); Bags/buckets (38.7%); OD (18.3%)
	Night	Bags/buckets (86.6%); Public (11.6%); Plot (1.5%); OD (0.3%)	Bags/buckets (92.8%); Public (7.2%)
Profile 3 **–**Toilet accessible at all times (34.3%)	Day	Plot (95.5%); Public (4.5%)	Plot (92.2%); Public (4.3%); Bags/buckets (3.4%)
	Night	Plot (70.6%); Bags/Buckets (29.4%)	Plot (51.3%); Bags/bucket (47.6%); Private (1.1%)
Profile 4 **–**Only in an emergency (11.3%)	Day	Private-shared (75.1%); Private (20.8%); Public (3.9%); Plot (0.1%)	Private-shared (68.9%); Private (19.2%); Bags/buckets (5.2%); Public (3.5%); OD (3.1%); Plot (0.1%)
	Night	Private-shared (32.3%); Bags/buckets (38.5%); Plot (14.7%); Private (14.4%)	Bags/Buckets (58.1%) Private-shared (22.4%); Plot (9.8%); Private (9.6%)
Profile 5 **–**No money/no access (15.6%)	Day	Bags/bucket (71.1%); Public (17.6%); OD (8.1%); Plot (3.1%)	Bags/bucket (90.4%); OD (9.4%) Plot (0.1%) Public (0.1%)
	Night	Bags/bucket (96.0%); OD (2.9%); Private-shared (1.0%); Plot (0.1%)	Bags/bucket (99.9%); OD (0.1%)

### Qualitative results

All women in the qualitative sample were asked to describe their primary methods of dealing with all short and long calls during the day and night and their reasons for adopting and using those strategies. These responses were used to help name, expand, and better define the common sanitation profiles from the LCA.

Based on simple frequencies of the qualitative data, 22% of the women in the qualitative sample fit the first sanitation utilization profile, i.e. using private, public, plot, or private-shared toilets during the day for both long and short calls and using bags/buckets for both long and short calls during the night. An additional 51% of the women in the sample could be described as fitting into the second sanitation utilization profile—utilizing a private, public, plot, or private-shared toilet during the day for long calls and some combination of bags/buckets/no facility for all other calls during the day and night. About 15% of the women in the sample could be described as following the third sanitation utilization profile—using a private, private-shared, or plot toilet for all calls during the day and night. Very few women in the qualitative sample (about seven percent) fit neatly into the fourth sanitation utilization category, i.e. women who primarily use a private, private-shared, public, or plot toilet for their long and short calls during the day and their long calls during the night and buckets for their short calls at night. Only 2 women (four percent) in the qualitative sample fit into the fifth sanitation profile—using bags/buckets/no facility for all long and short calls during the day and night.

#### Profile 1 –“Lack of security at night”

**Fig 3:**
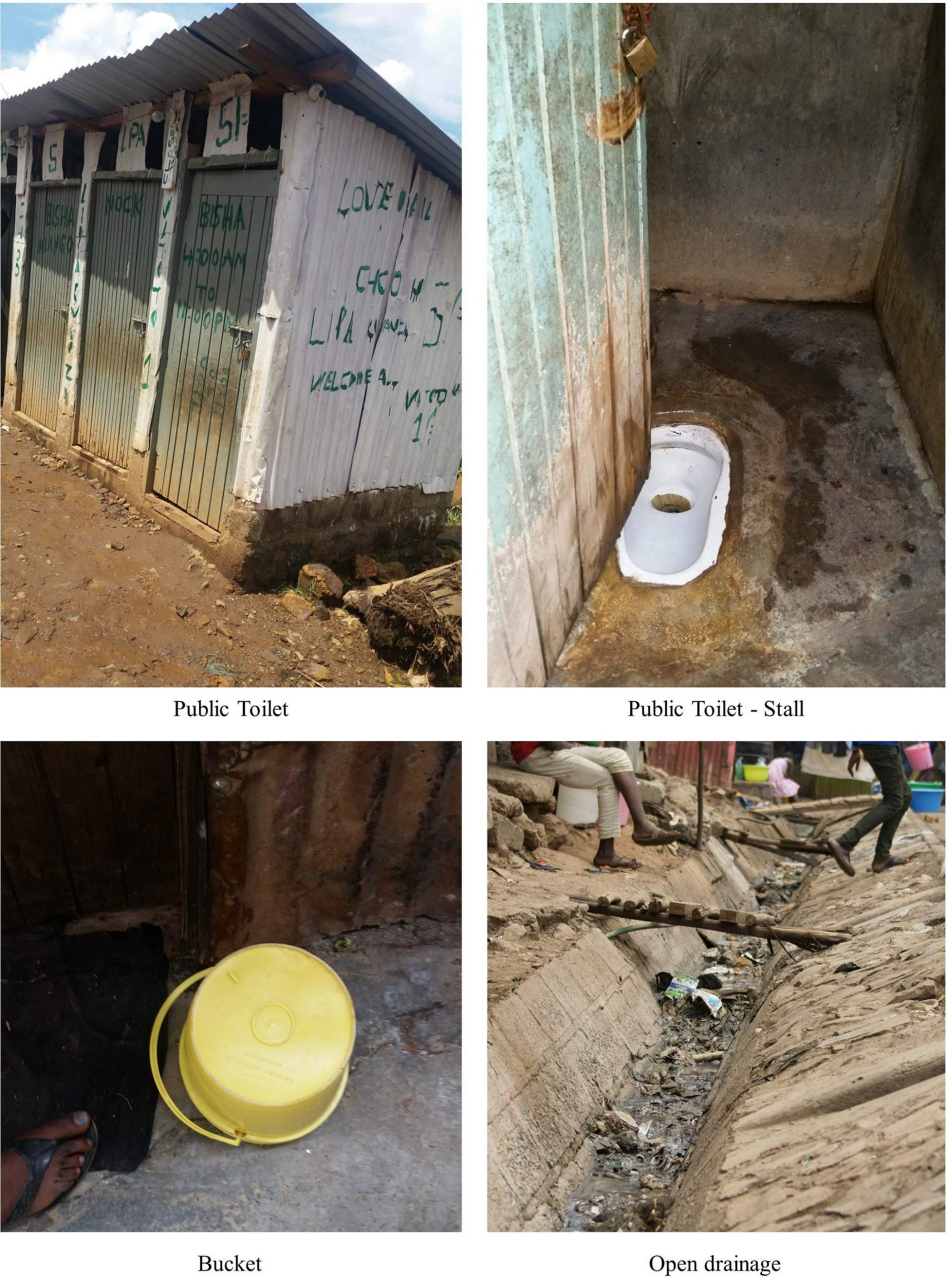
Example representation of SP1.

Women in this first profile (SP1) primarily utilize toilets (e.g. public, plot, or private-shared facilities) for long calls and short calls during the day and use bags/buckets/OD for long and short calls during the night. Women in this category reported using a toilet during the day for both long and short calls; however, almost all of the women in this profile reported using bags or buckets in the home at night to meet their sanitation needs because they could not go outside at night. Women in this profile cited “insecurity” in Mathare as the primary reason they could not go outside at night to access a toilet. For example, “at night, it’s this, whatever, bucket because at night, you cannot go outside. Often, maybe, you can get a person who can even rape you. You see? Now, people here fear the night because this is Mathare. It is the real Mathare, it’s not good.” (Sha, 4B). For this reason, this profile was labeled the “the lack of security at night” profile. Photos representing SP1 are shown in Fig 3.

#### Profile 2 –“Lack of funds and accessibility”

**Fig 4:**
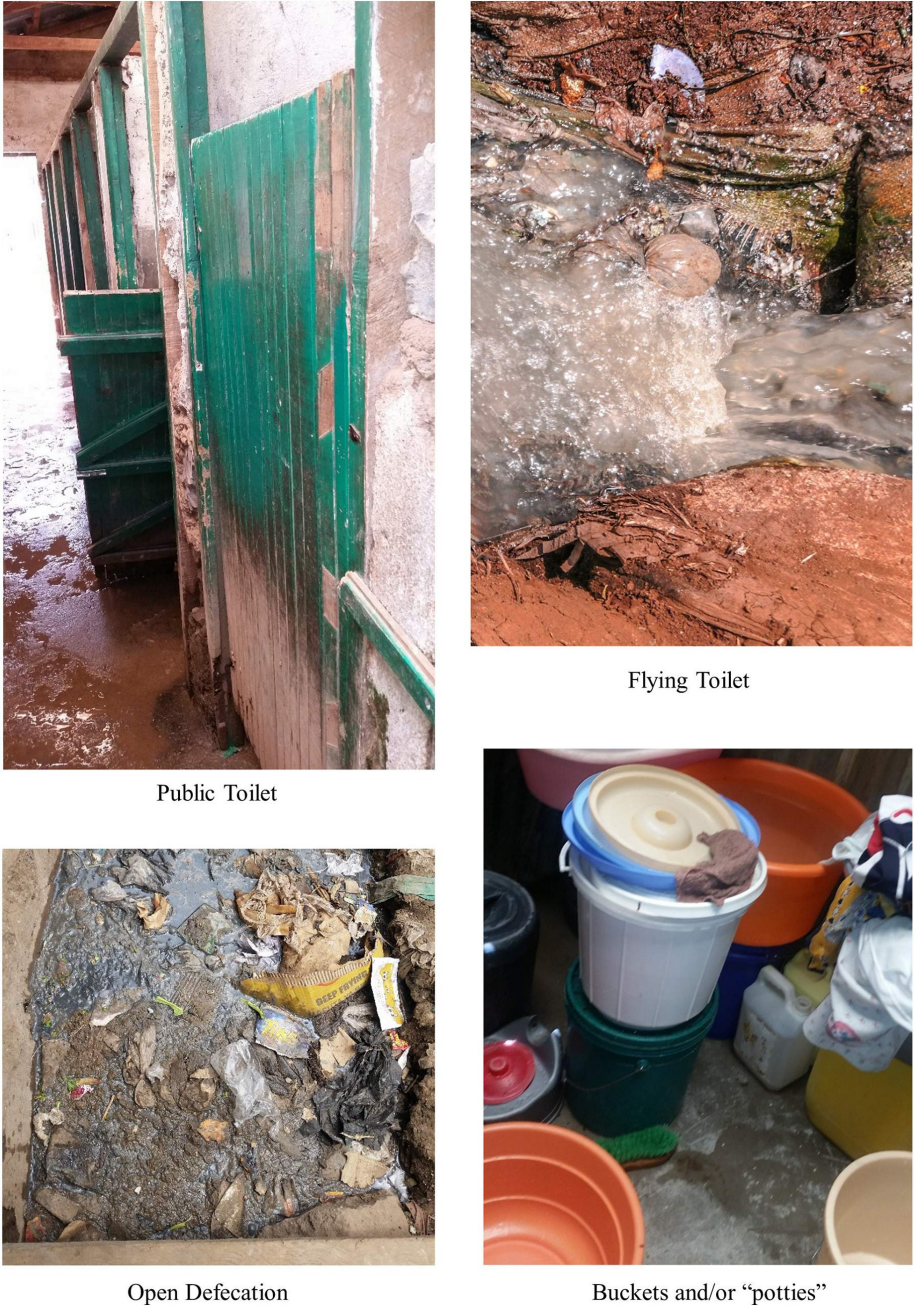
Example representation of SP2.

Profile 2 (SP2)—utilizing a private, public, plot, or private-shared toilet during the day for long calls and bags/buckets/no facility for all other calls during the day and night—was the most common sanitation category for women in the qualitative sample. What makes women in this profile different from those in the first sanitation profile, is that, in addition to not utilizing a toilet during the night for long or short calls due, in large part, to security concerns, they also did not visit a toilet for short calls during the day. Women cited two primary barrier to using a toilet for short calls during the day including difficulty paying the fees to use a public toilet, e.g., “if it is a short call, you won’t survive if you’re paying all the time. You urinate in a bucket, you go, you pour it in the drainage. But if it is a long call, you pay the 5 shillings to enter the toilet” (Sus, Kosovo) and/or distance to reach a toilet, e.g., “for short calls, we always use the bucket because we do not have a toilet near (Est, Mabatini). Thus, we labeled Profile 2 the “lack of funds and accessibility” utilization profile. Fig 4 illustrates a potential combination of sanitation options for SP2.

#### Profile 3 –“Toilet is accessible at all times”

**Fig 5:**
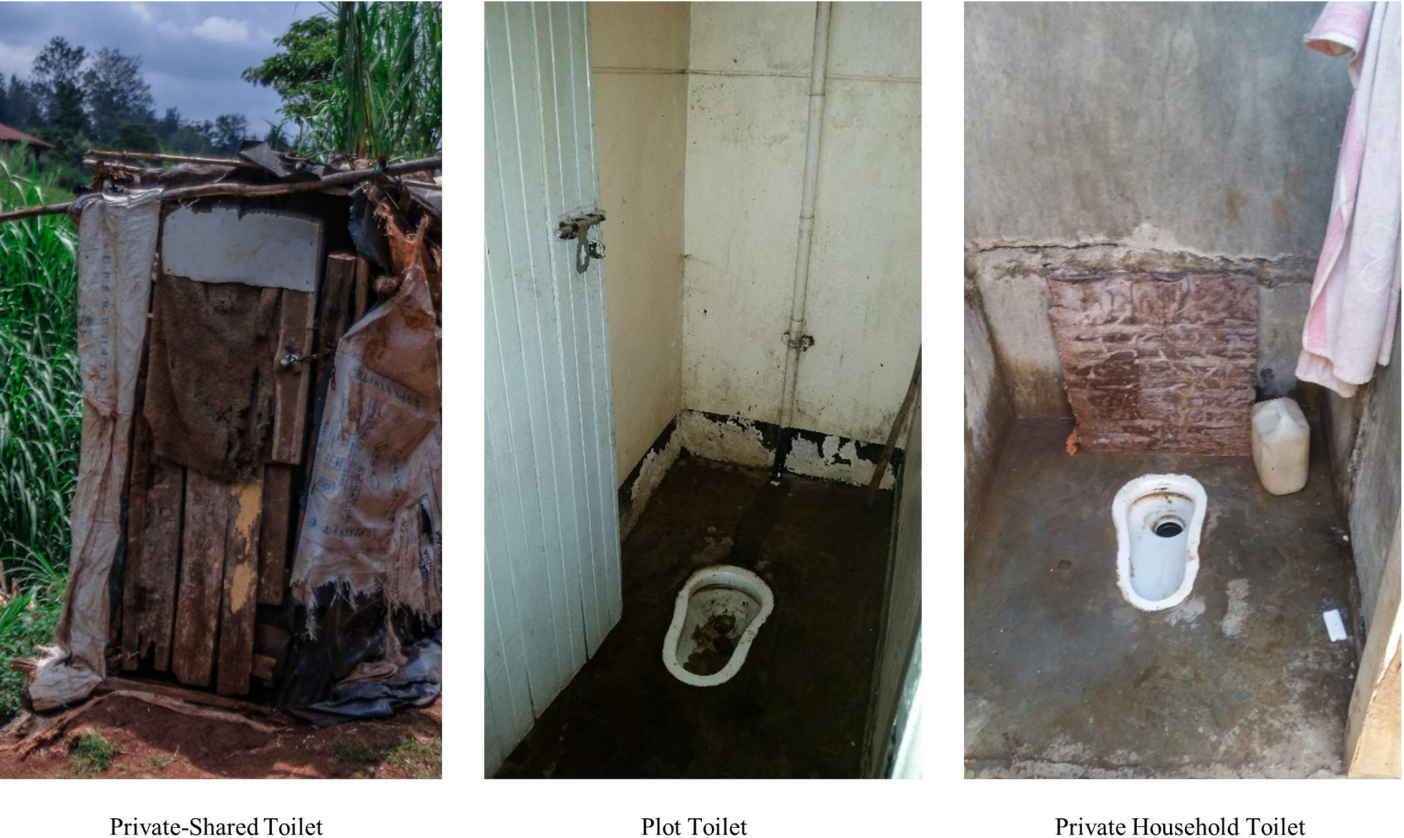
Example representation of SP3.

Women in this profile (SP3) reported that they had access to and utilized a private toilet, a private-shared facility, or a plot toilet for all calls during the day and night. There were only two women in the sample who had a private toilet connected directly to their home. The other women in this profile reported that their toilets were located within their building and, often, on their floor. Profile 3 was labeled the “toilet is accessible at all times” profile because it consists of women who, for the most part, have access to toilets which are safe and close by at all times during the day and night—making it possible for women to access them throughout a 24-hour period. Fig 5 shows some typical sanitation options for SP3.

#### Profile 4 –“Only in an emergency”

**Fig 6:**
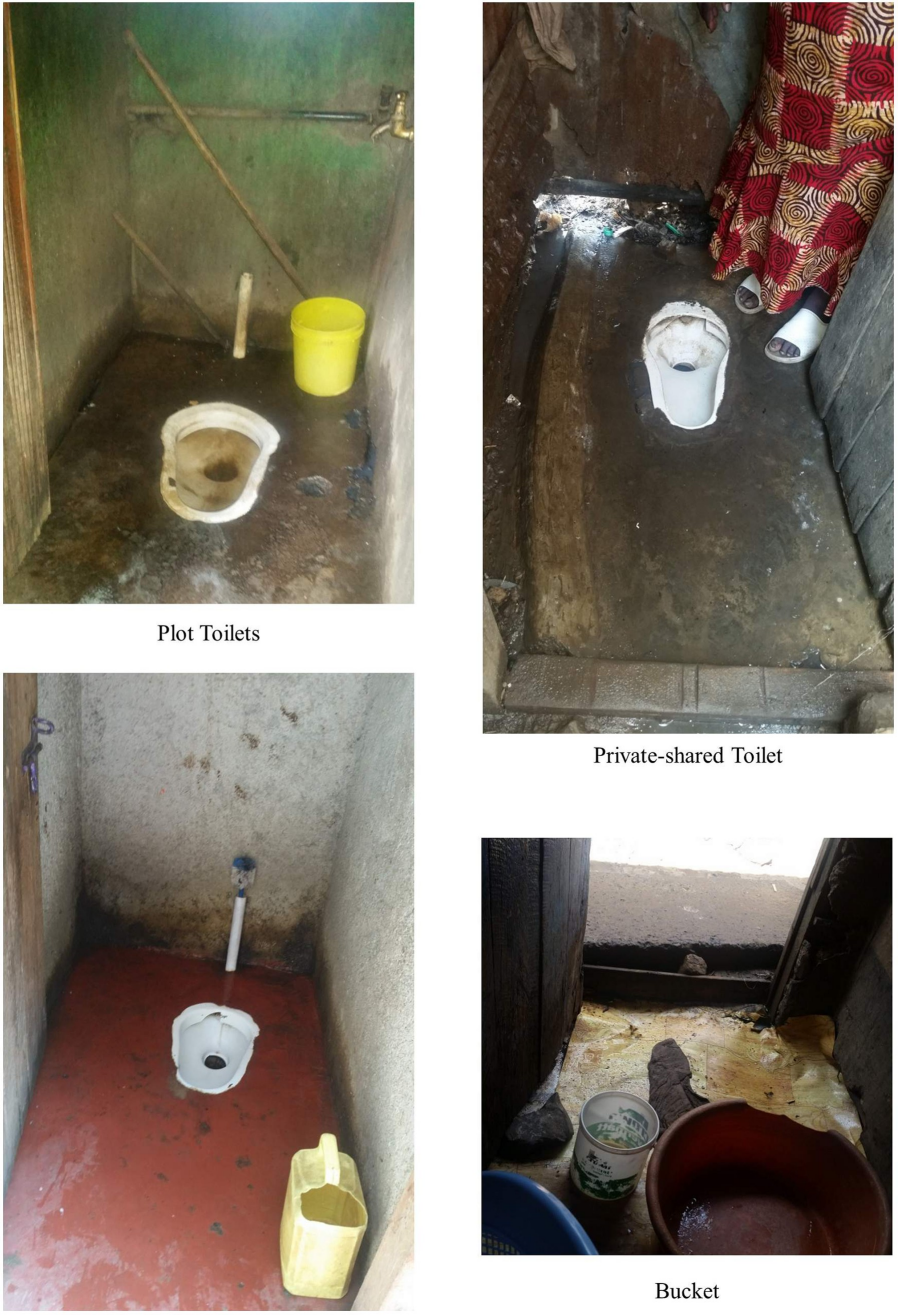
Example representation of SP4.

Women in the fourth profile (SP4) have a higher probability of using a toilet facility for long and short calls during the day and for long calls during the night and using a bucket for short calls during the night. This was not a common sanitation utilization strategy among women in the qualitative sample. About seven percent of the sample (4 women) fit clearly into this category. Most of the women in this profile stated that they had trained themselves not to urinate and/or defecate at night, i.e. “[at night] it is a must you constrict yourself, even if there is a toilet here [in the plot], often there is no security” (Hel Mabatini); however, they also stated that they would resort to going outside to use a toilet for long calls in an “emergency,” i.e., “if you are having stomach problems” (Eli Gitathuru; Eli Village 2). Therefore, sanitation utilization profile 4 was labeled the “only in an emergency” category. Fig 6 shows an example profile for SP4.

#### Profile 5 –“No money or no access”

**Fig 7:**
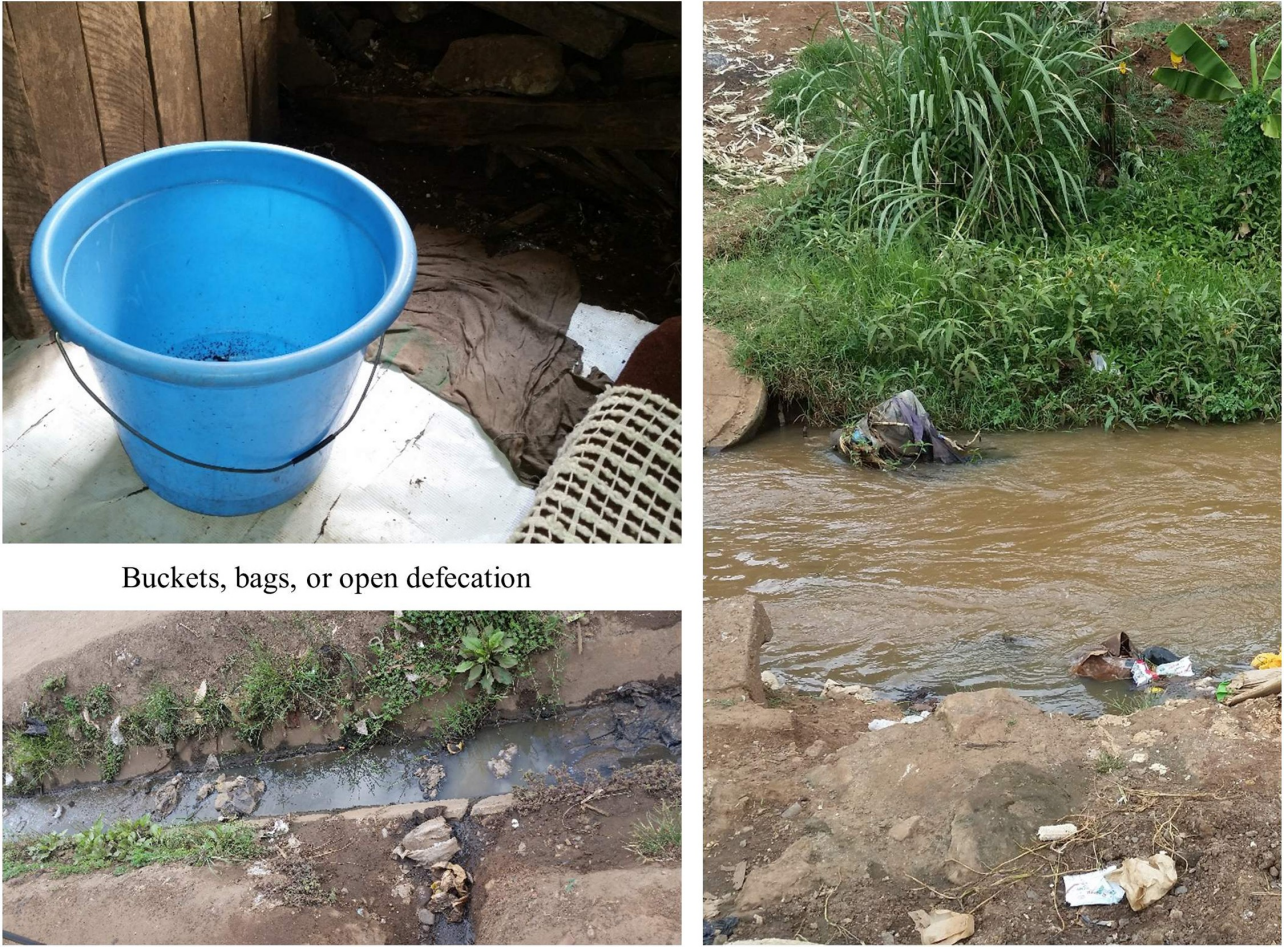
Example representation of SP5.

There were two women in the qualitative sample who reported using bags/buckets in the home for all calls during the day and night. These women stated that they used bags, buckets, and/or open defecation for all calls during the day and night, on a regular basis, because they could not afford to go to a public toilet and the toilets were far. These women talked about the expense of the public toilets being the primary barrier to access. For example, “for me, if I miss [to get money], it is a must I put a paper bag there… I cannot go to help myself…it is a must I just do this” (Ros, Gitathuru). Another woman stated that she is sometimes harassed at the toilets for not being able to pay, “like if you don’t have money, you beg…if they refuse, you just suffer… if I don’t have money, see, I will just hold it, I’ll come here [to the house] to this bucket” (Jan, Kosovo). In addition to having no money to go to the toilet, the women also expressed that the available toilets in the area were too far away; thus, this profile was labeled the “no money, no access” sanitation utilization profile. Photos for a typical SP5 profile are shown in Fig 7.

## Discussion

The purpose of this study was to explore women’s current sanitation practices in informal settlements in Nairobi, Kenya. First and foremost, results from this study suggest that women’s sanitation utilization in informal settlements in Nairobi is varied and quite complex—a finding that is consistent with previous qualitative studies that have indicated that women’s strategies for managing sanitation in informal settlements are complicated [26, 28, 30]. In particular, findings from this study suggest that the majority of women have different strategies for handling urine and feces and/or for managing their sanitation during the day versus during the night. Yet, despite the variations and complexity, findings from this study also suggest that there are some common sanitation utilization profiles that can better represent women’s current sanitation practices in this context.

Based on the quantitative LCA, there were five common sanitation utilization profiles among women in Mathare. These profiles help reflect general patterns of women’s sanitation utilization while still capturing variations in women’s strategies to handle urine and feces during the day and during the night. Profiles ranged from women who had access to and utilized toilets for all urine and feces disposal during the day and night to women who utilized unsafe methods of disposal for all urine and feces disposal.

One of the most important findings from this study was that women in four out of the five profiles (SP1, SP2, SP4, and SP5)—accounting for the majority of the women in the sample—regularly rely on “unsafe” forms of sanitation (e.g. bags, buckets, or open defecation) for at least one call (short and/or long) during the day and/or night. This is a central finding in light of other published estimates suggesting that only 6% of people in informal settlements in Nairobi, Kenya lack access to sanitation [32]. These findings suggest that women, despite having access to toilets for at least some portions of the day, are unable to use these facilities for one or more calls during the day and/or night for a variety of reasons.

Expanded sanitation coverage has been the emphasis of many sanitation policies and interventions in developing countries. It is only recently that scholars, policy-makers, and developers have started to recognize that availability of toilets does not necessarily equate to sustainable utilization of sanitation [37]. Goal 6 of the SDGs to “ensure availability and sustainable management of water and sanitation for all,” in particular, calls on researchers, developers, and decision-makers to pay special attention to the needs of women and girls. Data from this study provide some key insights into what those needs might be. Results from this study suggest that there may be a number of factors associated with sanitation utilization. For example, insecurity at night—a factor that has only recently materialized as an important factor in sanitation-related literature [16, 29, 38–40]—manifested as a critical issue for women in the sanitation profiles in this study.

Although the profiles developed in this study help capture general sanitation utilization patterns among women in informal settlements in Nairobi, findings from the qualitative data also suggest these profiles are probably not static. Many women may, and probably do, flow through a number of different profiles over the course of a week, month, or year. For example, a woman fitting the second profile, i.e. a woman who uses a public toilet for long calls during the day and then reverts to using bag/buckets in her home for short calls during the day, could very easily move into the first profile (using a public toilet for both long and short calls during the day) if she has extra money to use on sanitation. Recent literature suggests, however, that women may not only lack day-to-day funds to access sanitation—often because they lack control of the finances in the home—but may also lack sanitation-related decision-making power in the home to invest in or adopt new sanitation strategies [41, 42]. As more sanitation-related interventions are being piloted in informal settlements in Kenya, in general, and in Mathare, specifically, such as Sanergy’s Fresh Life toilets, Grand Challenge Canada’s funded Banza toilets, and/or National Youth Service’s (NYS) slum improvement project toilets, it seems critical to pay specific attention to the needs of women and gender-specific power relations in the home and how or if these interventions are affecting the sanitation profiles of women in these communities. It is important to ask, for example, whether these or future interventions: 1) meet the needs of women, and 2) help women who regularly rely on unhygienic sanitation management strategies such as bags, buckets, or open defecation during the night or day to overcome gender-specific barriers to adopt more hygienic sanitation management strategies.

In addition to the implications of this study with regard to women’s specific sanitation utilization in informal settlements in Nairobi, Kenya and for sanitation policies such as the SDGs, this study also has more general implications for research. Since access to basic sanitation was recognized in Target 7 of the Millennium Development Goals in 2000, there have been countless global- and local-levels efforts to quantify people’s access to sanitation all over the world. In fact, with the establishment of the Joint Monitoring Programme (JMP), there is yearly monitoring of global access to sanitation [1]. However, most measures of access to sanitation are based on survey responses to questions about participants’ *primary* toilet facility and/or method of urine/feces disposal [43]. Findings from this study indicate, however, that this common method of measuring sanitation utilization has failed to capture the variation and complexity of women’s sanitation utilization. By asking questions that explored women’s methods of dealing with short and long calls, separately, and their strategies during the day and during the night, this study was better able to capture the complexity of women’s sanitation utilization. While these methods and measures may not be important or relevant in all contexts, the results of this study suggest there is a need, at the very least, for sanitation researchers to develop appropriate methods and measures beyond standardized questions focused on individual’s *primary* methods of disposal.

Although this study yielded important results, it had a number of limitations. First, the small sample size limited our ability to keep the granularity of the sanitation variables in the models. In the survey women were asked many details about their sanitation methods/facilities including the type (e.g. flush, pour-flush, composting, bags, buckets, open defecation, pit latrine with slab, pit latrine without slab), where the urine/feces flows/is disposed (e.g. sewers, rivers, open drainages, septic tanks, pits), and technological features of toilet facilities (e.g. presence of superstructure, roof, doors, water for flushing and hygiene). For the purposes of these analyses these variables had to be collapsed into simpler variables with limited categories. Additionally, while the study used a stratified random sample, there are 200,000 residents in Mathare. It is unlikely that these 550 women represent the sanitation profiles of all women in the settlement.

## Conclusion

The findings from this study are important for sanitation researchers, developers and policy-makers. First, this is the first attempt to assess the complexity of women’s diurnal and nocturnal sanitation practices for short and long calls and to understand the underlying reasons behind their practices. Most quantitative efforts to enumerate people’s access to sanitation rely on questions about primary sanitation facilities/methods. This study illustrates a need for researchers to modify common measures of people’s sanitation utilization to capture the complexity of sanitation behavior, particularly in informal settlements. In addition, while many studies have shown that unimproved sanitation remains a serious issue in informal settlements, the results of this study indicate that the proportion of women reverting to unimproved sanitation is perhaps much higher than previously documented. Taking into account women’s actual sanitation practices and the reasons for those practices may have huge health and environmental implications. For example, almost all of the women in this study who reported using bags and buckets also reported emptying them into open drainage systems. People, particularly children, who are exposed to raw sewage in their environment, have a much greater risk of getting sick or dying from pathogen-related illnesses. In addition, there is a higher risk of large-scale outbreaks such as cholera and/or typhoid. Finally, many women reported using buckets and bags because they fear going outside at night. This suggests that toilet access for women is not only a function of availability, but of security, particularly in areas where crime rates and social disorganization are high. While there have been some recent studies that have attempted to empirically assess the relationship between access to sanitation and violence, there is a need for more studies that explore, not only the direct relationship between women’s access to sanitation and violence, but also the influence exerted by women’s perception of safety/security and fear of violence.

## Supporting information

S1 Survey Quantitative survey. (DOCX)

S1 Dataset Quantitative dataset. (XLS)

S1 Checklist COREQ-32-item checklist. (XLSX)
