# Supplementary material for: A mixed-methods study of women’s sanitation utilization in informal settlements in Kenya
Source: PLoS One. 2019 Mar 21;14(3):e0214114. doi: 10.1371/journal.pone.0214114 (PMC6428280; doi:10.1371/journal.pone.0214114)
Supplement: S1 Survey — (DOCX) [file pone.0214114.s001.docx]

| **SECTION 1: DEMOGRAPHICS**  *First, thank you so much for volunteering to participate in this study. Please try to answer all questions as truthfully and completely as possible. There are no right or wrong answers. If you are confused as any point during the survey, please feel free to ask me questions. Also, remember that this survey is voluntary. The information you provide is very important, but you are free to stop the survey at any time or to choose not to answer certain questions. I would like to start by asking a few questions about you.* | | | |
| --- | --- | --- | --- |
| **#** | **QUESTION** | **RESPONSE & INSTRUCTIONS** | **CODE** |
| **D1** | **How many people live in this household?** *Tafadhali unaweza kuniambia ni watu wangapi wanaishi hapa?* | [SPECIFY]  **Don’t know** ……………………..…………….……..  **No Response** ……………………………………...… | **8888**  **9999** |
| **D2** | **How old are you?**  *Uko na umri gani* | [SPECIFY]  **Don’t know** ……………………………..…………..  *Sijui*  **No Response** …………………………………..……  *Sina jibu* | **8888**  **9999** |
| **D3** | **What is the highest level of education that you have completed?**  *Umesoma mpaka kiwango gani* | **None** …………………………………………………  *Sijasoma*  **Some primary, but not completed** ……………...…  *nilisoma shule ya msingi, lakini sikukamilisha*  **Completed primary school** ………………………...  *nilikamilisha shule ya msingi*  **Some secondary, but not completed** ………………  *nilisoma shule ya sekondari, lakini si kukamilisha*  **Completed secondary** ………………………………  *nilikamilisha shule ya sekondari*  **Some higher education, but no degree** ……………  *nimesoma elimu ya juu* *lakini hakuna digrii*  **Completed a higher education degree** …………….  *Nilikamilisha shahada ya elimu ya juu*  **No Response** ………………..……….……………… | **1**  **2**  **3**  **4**  **5**  **6**  **7**  **9999** |
| **D4** | **Are you currently married or do you have a partner?**  *Je, umeolewa kwa sasa au una mpenzi wa kiume?* | **Currently married** ……………………….……..….  *kwa sasa nimeolewa*  **Living with a man, not married** ……………….…..  *naishi na mwanaume hatujaoana*  **Regular partner, live apart** ………………….…….  *mpenzi ,hatuishi pamoja*  **Not involved in a relationship** ……………….…….  *sina uhusiano wowote*  **Don't know**…………………..……………….……..  *Sijui*  **No Response** ………………..…………….………… | **1**  **2**  **3**  **4**  **8888**  **9999** |
| **D5** | **How many children do you have?**  *Uko na watoto wangapi?* | [SPECIFY]  **No Response** ……………………………..……….… | **9999** |

| **#** | | **QUESTION** | | **RESPONSE & INSTRUCTIONS** | | | | | **CODE** | |
| --- | --- | --- | --- | --- | --- | --- | --- | --- | --- | --- |
| **D6** | | **Do you work?**  *Je unafanya kazi?* | | **Yes** ………………………………..…………………  *ndio*  **No**……………….……………...……………………  *hapana*  **Don't know**………….…………..…………………..  *Sijui*  **No Response** ………………..……………………… | | | | | **1**  **0**  **8888**  **9999** | |
| **D7** | | **Do you have a business?**  *Je uko na biashara?* | | **Yes** ………………………………..…………………  *ndio*  **No**…………………………...…………..……………  *hapana*  **Don't know**…………………..……….……………..  *Sijui*  **No Response** ………………..……….……………… | | | | | **1**  **0**  **8888**  **9999** | |
| **D8** | | **What would you estimate your household monthly income to be (Ksh)?**  *Mapato ya kila mwezi ya familia kwa ujumla ni kama pesa ngapi?* | | [SPECIFY]  **Under 5,000 Ksh per month**  **Between 5,000-10,000 Ksh per month**  **Between 11,000-15,000 Ksh per month**  **Over 15,000 Ksh per month**  **Don’t know** ………………………..………….……..  *Sijui*  **No Response** ……………………………..……….… | | | | | **1**  **2**  **3**  **4**  **8888**  **9999** | |
| **SECTION 2: PRIMARY TOILET FACILITIES**  *Thank you so much for participating in the study. I would now like to start asking questions related to your personal sanitation practices, i.e. both long calls (defecation) and short calls (urnition) during the day and during the night.* | | | | | | | | | | |
| **T0**  ******* | | **Please describe how you relieve yourself during the day and night for long call (defecation) and short call (urination).**  *Tafadhali, eleza unavyojisaidia usiku na mchana kwa haja kubwa na haja ndogo.*  ***[SPECIFY]*** | **Day/Urination**  **Mchana / Haja Ndogo** | | **Night/Urination**  **Usiku / Haja Ndogo** | | | | | |
|  |  |  | **Day/Defecation**  **Mchana / Haja Kubwa** | | **Night/Defecation**  **Usiku / Haja Kubwa** | | | | | |
| **#** | **QUESTION** | | **RESPONSES &**  **INSTRUCTIONS** | | | **DAY** | | **NIGHT** | | |
|  |  |  |  |  |  | **Long** | **Short** | **Long** | | **Short** |
| **T1** | **What kind of [toilet, site for defecation or method of disposal] do you usually use during the [*day/night]* for [*long call/short call]*?**  W*ewe hutumia njia/choo cha aina gani ya kujisaidia mchana na usiku kwa haja kubwa na haja ndogo?* | | **Flush** ……………..……………. *choo cha maji*  **Pour Flush** …………...………... *choo cha kumwaga maji mwenyewe*  **Ventilated improved pit latrine (VIP)** *choo cha shimo*  **Pit latrine with slab** …..………. *choo cha shimo cha simiti*  **Pit latrine without slab/open pit**  *choo cha shimo bila simiti/shimo la wazi*  **Bucket toilet** …….………..…… *choo cha ndoo/kasuku*  **Hanging toilet**  *choo juu ya mtaro/mto*  **Plastic bag/Newspaper** …..….... *mfuko wa plastiki/karatasi/gazetti*  **No facility, bush or field** …….... *hakuna kituo, kichaka au shamba*  **Bathroom/shower** ……………..  *Bafu*  **Other (specify)** ………………...  *nyingine (taja)*  **No Response** ……….…..……… | | | **1**  **2**  **3**  **4**  **5**  **6**  **7**  **8**  **9**  **10**  **9999** | **1**  **2**  **3**  **4**  **5**  **6**  **7**  **8**  **9**  **10**  **9999** | **1**  **2**  **3**  **4**  **5**  **6**  **7**  **8**  **9**  **10**  **9999** | | **1**  **2**  **3**  **4**  **5**  **6**  **7**  **8**  **9**  **10**  **9999** |

| **#** | **QUESTION** | **RESPONSES &**  **INSTRUCTIONS** | **DAY** | | **NIGHT** | |
| --- | --- | --- | --- | --- | --- | --- |
|  |  |  | **Long** | **Short** | **Long** | **Short** |
| **T2** | **Approximately how many individuals (including you and your family) use this toilet on a regular basis?** *Takriban watu wangapi (pamoja na wewe na familia yako) hutumia hichi choo mara kwa mara?* | [SPECIFY] ………………………..  **Don’t know** ………………...……..  **No Response** ………………...…… | **____**  **8888**  **9999** | **____**  **8888**  **9999** | **____**  **8888**  **9999** | **____**  **8888**  **9999** |
| **T3** | **Can any member of the public use this toilet?**  *Je mtu yeyote anaweza kutumia hichi choo* | **Yes** ……………….…………..……  *ndio*  **No** …………………….…..……….  *hapana*  **Don't know** …………….…..……..  *Sijui*  **No Response** …………….…..…… | **1**  **0**  **8888**  **9999** | **1**  **0**  **8888**  **9999** | **1**  **0**  **8888**  **9999** | **1**  **0**  **8888**  **9999** |
| **T4** | **Is this toilet located at your place of work?**  *Je hichi choo kiko mahali ambapo unafanya kazi?* | **Yes** ……………….…………..……  *ndio*  **No** …………………….…..……….  *hapana*  **Don't know** …………….…..……..  *Sijui*  **No Response** …………….…..…… | **1**  **0**  **8888**  **9999** | **1**  **0**  **8888**  **9999** | **1**  **0**  **8888**  **9999** | **1**  **0**  **8888**  **9999** |
| **T5** | **Is this a plot toilet?**  *Je hichi choo kimo plotini yako?* | **Yes** ……………….…………..……  *ndio*  **No** …………………….…..……….  *hapana*  **Don't know** …………….…..……..  *Sijui*  **No Response** …………….…..…… | **1**  **0**  **8888**  **9999** | **1**  **0**  **8888**  **9999** | **1**  **0**  **8888**  **9999** | **1**  **0**  **8888**  **9999** |
| **T6** | **Do you usually go outside your home to use this toilet/method?**  *Kawaida, unabidi kutoka nje kutumia hichi choo/njia?* | **Yes** …..…………………….………  *ndio*  **No** ……..…………….…………….  *hapana*  **Don't know** ……..……….………..  *Sijui*  **No Response** ……………….…..… | **1**  **0**  **8888**  **9999** | **1**  **0**  **8888**  **9999** | **1**  **0**  **8888**  **9999** | **1**  **0**  **8888**  **9999** |
